# Use of pulse pressure variation and stroke volume variation in spontaneously breathing patients to assess dynamic arterial elastance and to predict arterial pressure response to fluid administration

**DOI:** 10.1186/cc13341

**Published:** 2014-03-17

**Authors:** M Cecconi, MI Monge García, M Gracia Romero, J Mellinghoff, F Caliandro, RM Grounds, A Rhodes

**Affiliations:** 1 St George's Hospital, London, UK; 2 St George's Healthcare NHS Trust and St George's University of London, UK

## Introduction

Dynamic arterial elastance (Eadyn), defined as the pulse pressure variation (PPV) to stroke volume variation (SVV) ratio, has been suggested as a predictor of the arterial pressure response to fluid administration. Rather than a steady-state assessment, Eadyn depicts the actual slope of the pressure-volume relationship providing a dynamic evaluation of the arterial load. So the higher the Eadyn value, the more likely arterial blood pressure is to improve after fluid challenge (FC). The aim of this study was to assess the effectiveness of Eadyn, measured non-invasively in preload-dependent, spontaneously breathing patients.

## Methods

Patients admitted postoperatively and monitored with the Nexfin monitor were enrolled in the study. Patients were included if they were spontaneously breathing and had an increase in cardiac output ≥10% after a FC. They were classified according to the increase in mean arterial pressure (MAP) after FC into MAP-responders (MAP increase ≥10%) and MAP-nonresponders (MAP increase <10%). Eadyn was calculated from the PPV and SVV values obtained from the monitor.

## Results

**Figure 1:**
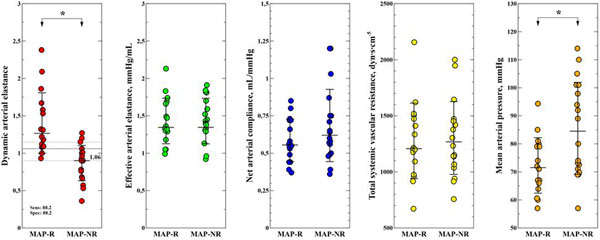
**Distribution of individual arterial load parameters at preinfusion time**.

A total of 34 FCs from 26 patients were studied. Seventeen FCs (50%) induced a positive MAP response. Preinfusion Eadyn was significantly higher in MAP-responders (1.39 ± 0.41 vs. 0.85 ± 0.23; *P *= 0.0001) (Figure 1). Preinfusion Eadyn predicted a positive MAP response to FC with an area under the ROC curve of 0.92 ± 0.04 of SE (95% CI: 0.78 to 0.99; *P *< 0.0001). A preinfusion Eadyn value ≥1.06 (grey zone: 0.9 to 1.15) discriminated MAP-responders with a sensitivity and specificity of 88.2% (95% CI: 64 to 99%).

## Conclusion

Non-invasive dynamic arterial elastance, defined as the PPV to SVV ratio, predicted the arterial pressure increase to fluid administration in spontaneously, preload-dependent patients.

